# Phosphodiesterase Type 5 Inhibitors as Chemical Hazards in Honey and Honey-Based Products

**DOI:** 10.3390/molecules31152681

**Published:** 2026-07-31

**Authors:** Joanna Wojtacka

**Affiliations:** Department of Veterinary Public Health, Faculty of Veterinary Medicine, University of Warmia and Mazury in Olsztyn, 10-718 Olsztyn, Poland; joanna.wojtacka@uwm.edu.pl

**Keywords:** honey, aphrodisiac honey, sildenafil, tadalafil, PDE-5 inhibitors, chemical hazard, food safety, RASFF

## Abstract

The processing of bee products has recently become more frequent due to the rapidly growing market and demand for healthy, natural, and functional food. Therefore, bee products, and above all honey, while maintaining the reputation of healthy and natural foods, become carriers of various additives. Their consumption without proper control, or not declaring their presence in the composition, may pose a significant hazard to human health and life. This study was based on data mining with the use of all accessible RASFF records. They were formatted to analyze all chemical hazards that have occurred in bee honey and have been noted by EU countries to support swift reaction by food safety authorities since 2014. Within 10 food categories presenting 13,295 records, 93 records applied to bee products, including 79 records that referred to honey, 65% (*n* = 51) of which related to chemical hazards. PDE-5 inhibitors, i.e., sildenafil and tadalafil, were identified 14 times during the last 6 years and were the second most numerous group of hazards in honey and honey-based products after VMPs. The periodically published results of market sample tests and the continuing trend of RASFF notifications of these compounds in honey and honey-based products may suggest an iceberg phenomenon that requires frequent and thorough inspections.

## 1. Introduction

Phosphodiesterase type 5 (PDE-5) inhibitors are classified into three generations: first-generation drugs, such as sildenafil and vardenafil, second-generation drugs, such as tadalafil, and third-generation drugs, such as avanafil [1]. Sildenafil citrate (Viagra^®^) and tadalafil (Cialis^®^) are the two most common drugs based on PDE-5 inhibitors used around the world [2].

Sildenafil shows primarily its efficacy in the treatment of erectile dysfunction (ED) and pulmonary arterial hypertension (PAH); however, it can also have beneficial effects in each organ, such as the heart, liver, kidney, brain, and intestines [3]. For ED, the typical dosage of sildenafil-based medicines is 25, 50 or 100 mg once a day. Sildenafil treatment-related adverse events are headache, facial flushing, dyspepsia, dizziness, nasal congestion, abnormal vision, and palpitation. They can also include back pain, influenza-like syndrome, rash, vomiting, diarrhea, cardiac arrhythmia, and hypersensitivity reactions [4].

Tadalafil is commonly used for the treatment of ED and benign prostatic hyperplasia (BPH). It also shows positive effects in PAH and different cardiovascular diseases [5]. In the case of tadalafil-based medicines prescribed for ED, the typical dosage is 10 or 20 mg once a day. Common adverse effects after tadalafil treatment are headache, dyspepsia, myalgia, flushing, and nasal congestion. In rare cases, serious adverse reactions like sudden vision loss [6] or postorgasmic illness syndrome (POIS) [7] may occur. The adulteration of food or food supplements with PDE-5 inhibitors and their analogs is extremely dangerous for consumers due to their unknown efficacy and toxic effects [8]. Bee honey, when placed on the market as honey or used in any product intended for human consumption, should not have any food ingredient added to it, including food additives, nor should any other additions be made other than honey [9]. All additions classify honey as (a) honey-based products, i.e., aphrodisiac honey, or (b) an ingredient of the food supplement marketed in dose form, such as capsules, pastilles, tablets, pills, sachets of powder, ampoules of liquids, drop-dispensing bottles, and other similar forms of liquids and powders designed to be taken in measured small unit quantities [10], i.e., in the case of ED. In both procedures, honey is blended or accompanies unauthorized or undeclared PDE-5 inhibitors, which is against food binding law in the EU [11]. To perform international notification about these kinds of hazards to human health deriving from food or feed that occur on the territory of the EU, the Rapid Alert System for Food and Feed (RASFF) was established [11]. The register of the notifications introduced to the system by the competent authorities is open to the public and functions under the name of RASFF Window [12].

The aim of the study was to verify the frequency and learn the characteristics of the chemical hazards occurring due to adulteration with PDE-5 inhibitors in honey and honey-based products marketed and sold in the territory of the EU and being the subject of international trade based on the official controls conducted in the Member States and border controls, the results of which are registered in RASFF.

## 2. Results

Data mining of the records obtained from the RASFF Window [12] showed the presence of honey, either as a natural bee product or ingredient of food products, as a source of different chemical hazards in five food categories, i.e., cereals and bakery products (3/1974 records), confectionery (1/733 records), dietetic foods, food supplements and fortified food (8/2308 records), honey and royal jelly (38 hazards described in 37/53 records), and other food products/mixed (3 hazards described in 2/1227 records). The difference between the number of hazards (*n* = 53) and records (*n* = 51) occurred because 2 hazards overlap within the same notifications. The remaining five food categories reported notifications about honey, i.e., milk and milk products, non-alcoholic beverages, nuts, nut products and seeds, prepared dishes and snacks, soups, broths, sauces and condiments but applied to hazards different than chemical origin.

**Figure 1:**
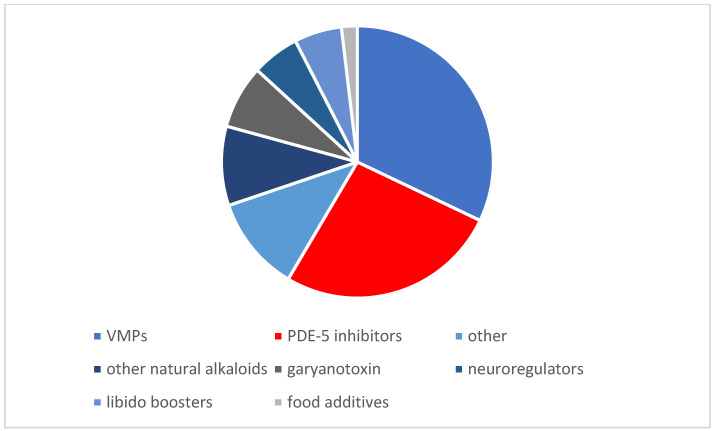
Distribution of chemical hazards in honey and honey-based products based on RASFF (2014–June 2026) records [12].

For the purposes of this analysis, the identified chemical hazards were divided into 8 groups. Honey turned out to be the carrier of numerous chemical hazards coming from the environment, i.e., various Veterinary Medicinal Products (VMPs), (*n* = 17), grayanotoxin (*n* = 4), other natural alkaloids like oxymatrine and matrine (*n* = 5). The chemical hazards notified came also from honey processing and blending with other components, i.e., PDE-5 inhibitors like sildenafil and tadalafil (*n* = 14), neuroregulators like tetrahydrocannabinol (THC), cannabidiol (CBD) and *Hericium erinaceus* (*n* = 3), libido boosters like *Epimedium* spp. or *Eurycoma longifolia* (*n* = 3), and food additives like tartrazine (E102), sunset yellow FCF (E110), allura (E129), (*n* = 1). There were also other chemical hazards coming from rusty containers or origins from countries with no residue control and presence of the allergen lactose (*n* = 6), Figure 1.

PDE-5 inhibitors were represented by sildenafil and tadalafil and were the second most numerous group of notifications related to honey. Both pharmacological agents were declared in 3 of 5 food categories each time as unauthorized or undeclared and detected in honey, honey-based products, and food supplements. Distribution of all chemical hazards in individual food categories is given in Table 1.

**Table 1:** Characterization of different chemical hazards, including PDE-5 inhibitors, in different food categories in honey and bee honey-based products according to official notifications (records) by EU supervising bodies [12].

Food/Feed Category	Number of Hazards	Type of Chemical Hazard	Hazard Characterization
Cereals and bakery products	2	other	origin from the country with no residue control
	1	food additives	breakfast cereals with honey containing too high levels of tartrazine (E102), sunset yellow FCF (E110), allura (E129)
Confectionery	1	other	desert containing honey from the country with no residue control
Dietetic foods, food supplements and fortified food	**7**	PDE5 inhibitors	undeclared sildenafil and tadalafil in honey, honey paste, honey-based products, sticks of aphrodisiac honey, honey-based food supplement
	1	neuroregulators	unauthorized CBD in honey
Honey and royal jelly	17	VMPs	semicarbazide, streptomycin, metronidazole, chloramphenicol, sulfonamides, paracetamol, ciprofloxacin, enrofloxacin and trimethoprim, tetracyclines, tylosine, dihydrostreptomycin, nitrofuran (semicarbazide), bacteriostatic drug Dapson in honey sulfadiazine, sulfadimidine, sulfamethoxazole, trimethoprim, coumaphos, acrinathrin, T-fluvalinate in comb honey dimetridazole in blend of organic honeys
	**5**	PDE5 inhibitors	unauthorized sildenafil and tadalafil in honey, in herbal honey based mixed paste, in honey product, in honey-based products
	5	natural alkaloids	oxymatrine and matrine in honey
	4	grayanotoxin	grayanotoxin in honey
	3	other	organic honey stored in rusty containers, allergen (lactose) in honey
	2	libido booster	*Eurycoma longifolia* in honey
	2	neuroregulators	tetrahydrocannabinol (THC), cannabidiol (CBD) and *Hericium erinaceus* in honey
Other food products/mixed	2	PDE5 inhibitors	sildenafil in honey paste, honey-based paste with herbs
	1	libido boosters	epimedium in honey-based paste with herbs

All notifications related to PDE-5 inhibitors in honey and honey-based products came from the years 2021–2026, Figure 2.

**Figure 2:**
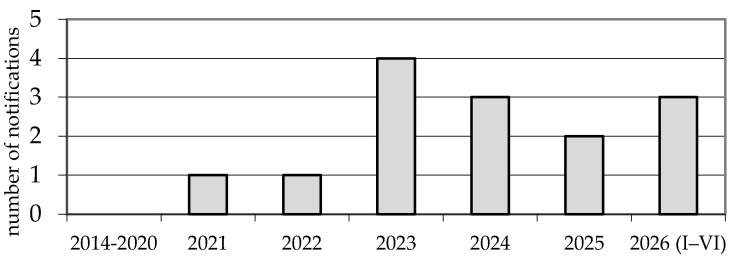
Number of notifications concerning PDE-5 inhibitors in honey and honey-based products introduced to RASFF from 2014 till June 2026.

Detection and further analyses of the products and supplements suspected of posing a hazard due to the presence of erectile substances in their composition were the result of routine controls and complaints received from the consumers. Among the notifications verified, five included the results of analytical tests, as shown in Table 2.

## 3. Discussion

Honey consumption has increased in recent years, and the European honey consumer uses honey mainly to flavor drinks, cakes, fruit, and seeds, for breakfast, as a healthy sweetener, and for direct consumption [13]. In the consumer’s perception, bee products, including honey, represent food of the highest quality. Due to its natural origin and complex chemical composition, honey is considered a product that improves vitality. It is a nutritious, healthy, and natural food with numerous health-promoting and supporting properties [14]. The addition of honey makes the product more credible and makes it appear healthy and safe.

Bee honey has been present in folk medicine for centuries. It was used to treat different health problems but also as a sexual stimulator. In this context, aphrodisiac honey, also known as mad honey, shows its properties thanks to the grayanotoxin that is collected by bees via the nectar of the rhododendron plants [15]. Botanical origin directly indicates the Asiatic or North American origin of mad honey. The presence of this toxin is controlled for, which is in line with the presented analysis of RASFF notifications, revealing only 4 cases of honey with grayanotoxin, 3 from Nepal and 1 from Singapore. Nevertheless, commercial honey can be described as safe because if there is any contamination, mass production dilutes the toxin quantities [16]. The sale and use of mad honey as an aphrodisiacal medicament in a modern setting is continuously growing, suggesting its popularity at a global level [17]. Nowadays, the term “aphrodisiac honey” has expanded to include honey and honey-based products enriched in pharmaceuticals during manufacturing. According to analysis of RASFF records, the most common drugs detected in honey and honey-based products were sildenafil and tadalafil, which are prescription drugs belonging to a different group than VMPs. They are prohibited for use in food products and food supplements in the EU [18].

Honey, as a product of animal origin, is incorporated into national programs for the official control and monitoring of hazards that can appear in food and pose risk to human health and life [19,20,21]. Originating from the environment, apiary management, and manufacturing, chemical hazards are the major risk related to honey [22,23]. The analysis conducted in this study indicated residues of VMPs as the most numerous groups of hazards in bee honey subjected to official controls, which is line with the results of the analysis of RASFF notifications from a five-year period [24]. However, this report showed drugs for ED as the third most frequent hazard in honey, while reviewing all RASFF records from 2014 till June 2026 indicated PDE-5 inhibitors were the second most frequent chemical hazard in honey, with three notifications in the first six months of 2026 compared to two notifications in the whole of 2025. These findings may indicate a rapid increase in the number of honey-based products adulterated with PDE-5 inhibitors, considering the fact that official controls are carried out randomly and bee products are not the largest group controlled in national residue monitoring programs. Moreover, although the levels of sildenafil and tadalafil confirmed in official laboratories seem low or within the scope of the doses recommended for ED, the dosage of the honey-based product indicated by the manufacturer may lead to a significant overdose of the drug. The daily dose of the honey-based product described in one of the notifications was one sachet (20 g) per day, which means that one intake was equal to the dose of sildenafil twice that high as the highest recommended. The sachet of this product also contained tadalafil at a dose between the lowest and highest recommended. The presence of this type of product on the market should be considered highly alarming, especially since cases of serious adverse reactions after consuming aphrodisiac honey were reported [25,26,27]. The risk of overdosing PDE-5 inhibitors in honey-based products also remains high due to the incorrect dosage indication or the lack of information on the unauthorized ingredient. The consumer taking the risk of consuming illegal food products without medical consultation is unable to estimate the basic risk related to co-occurring diseases or medications taken. PDE-5 inhibitors intake may carry additional health risks, especially in patients taking medications such as nitrates or α-blockers, potent cytochrome P450 3A4 inhibitors, including erythromycin, clarithromycin, ketoconazole, itraconazole, and HIV protease inhibitors [28]. There are numerous drugs that interact with sildenafil and tadalafil, including those that cause devastating adverse reactions [29]. The risk is also associated with simultaneous consumption of alcohol or certain types of conventional food. Concomitant ingestion of alcohol and PDE-5 inhibitors increases the risk of PDE-5 inhibitor-associated complications, including vasodilation symptoms, as well as seldomly observed life-threatening complications [30]. It is also recommended to avoid drinking pomelo and grapefruit juices while taking PDE-5 inhibitors due to physiochemical interactions that result in changes in the systemic exposure of the pharmacological substance [31]. Moreover, it is likely that patients with ED tend to consume multiple dietary supplements available, thereby exposing themselves to highly elevated cumulative levels [32] that may be impossible to assess since it happens that samples of the same commercial brand are positive for the PDE-5 inhibitors with varying concentrations in each sachet [33]. Although the analysis of RASFF notifications revealed adulteration of honey and honey-based products with sildenafil and tadalafil, there is research showing honey product adulteration with other PDE-5 inhibitors, i.e., vardenafil [32,34]. However, both the analysis of the records from the RASFF Window and the results published elsewhere indicate sildenafil is the major contaminant [32,33,34]. The result of 17.7% adulterated samples of honey or honey-based products obtained through RASFF Window reflects the food-borne hazards that have been already reported in the study based on the analysis of the samples purchased on the market, where 12% of the samples were found positive [33]. The results published by other authors, i.e., 38.5% [32] or 55% positive for PDE-5 inhibitor samples obtained from honey-based products purchased from medicine and spice shops and on social media [34], lead to the conclusion that food fraud in the form of honey and honey-based product adulteration with PDE-5 inhibitors fits into the iceberg model, where increasing awareness and proactiveness can be seen through the lens of digging deeper into the problem and discovering the levels of the problem that are “below the waterline” [35]. It indicates the chemical hazard posed by unauthorized and undeclared PDE-5 inhibitors is a real concern and must be monitored obligatorily not only upon consumer complaint or random sampling but also as a part of targeted monitoring, especially since there are several analytical methods designed for the detection of active pharmaceutical ingredients. These are high-performance liquid chromatography (HPLC), gas chromatography (GC), and mass spectrometry (MS), and new techniques have also been developed. They are aimed at screening honey-based food supplements adulterated with PDE-5 inhibitors and allow not only sample classification but also quantification of adulterants [35].

This research has some limitations. They are related to the source, which was a database of records implemented internationally by the EU countries and which can be described as a passive surveillance database. Routine reporting usually results in missed incidents, unknown denominators, and underreporting, issues that render some results incomplete in terms of details, i.e., analytical results. The food sample limit allocation scheme is based on a cascading (top-down) allocation of central limits and may differ in each member country based on previous results obtained and national programs of food safety monitoring. This means that not every batch of food undergoes official control. The results obtained from a database like RASFF can serve as indicators for further in-depth research and to raise awareness. Recommendations for further actions include descending and ascending activities, carried out by the official regulatory bodies responsible for public health. It would be recommended to make official screenings of honey and honey-based products coming from outside the EU, targeted specifically at PDE-5 inhibitors to assess the real risk level. In relation to public health, information campaigns about the possible side effects of falsely health-promoting products could prevent the undesirable effects of the consumption of adulterated food and supplements.

## 4. Materials and Methods

The research was based on the analysis of a database that came from the publicly available tool called RASFF Window [12]. Of 37 product categories included in RASFF, 10 final categories were selected based on the automatic search with the subject defined as “honey”, with a total of 13,295 records and 14 variables. The variables were given by the RASFF search tool automatically, and they included reference number, food category, type, subject, date, notifying country, classification, risk decision, distribution, for attention, for follow-up, operator, origin, and hazards. To eliminate other bee products, i.e., propolis from the automatic search database, some variables were added manually. They included the type of bee product, the method of use (raw honey, honey-based), and the exact type of hazard. From this group, 93 records were selected, with notifications describing hazards in food products that were bee products or contained bee products. Finally, 14 records were classified as non-related to honey, and 79 records related to honey were analyzed. Non-chemical hazards, i.e., larvae and moths in nut products with honey, were noted in the following 28 records. Due to the limitations of the MS Excel environment, the data set was divided into worksheets and subjected to a data segregation process. The actual data mining process was carried out using the built-in mathematical and statistical functions of MS Excel. Only those records that related to chemical hazards, including hazards overlapping in the same records (notifications), were further analyzed. They were divided into eight groups based on the type of hazard: VMPs, PDE-5 inhibitors, other natural alkaloids, grayanotoxin, natural neuroregulators and libido boosters, food additives, and others. The data sheet devoted to PDE-5 inhibitors was processed and enriched with information coming from the more in-depth search aimed at analytical results.

The data analyzed came from the period from February 2014 to June 2026.

## Figures and Tables

**Table 2:** The levels of PDE-5 inhibitors in bee honey and honey-based products analyzed as the result of the official controls [12].

Origin of the Sample	Intake Recommended by the Manufacturer	Pharmaceutical Ingredient Detected	Analytical Result	Unified Results
Honey-based food supplement from Turkey	1 measuring spoon (12 g)	sildenafil	6.17 mg/g	6.17 mg/g
Honey-based food supplement from Thailand	1 sachet/day (20 g)	tadalafil sildenafil	0.83 mg/g 9.57 mg/g	0.83 mg/g
Honey from Malaysia	na *	sildenafil	4.28 mg/g	4.28 mg/g
Honey product from the Syrian Arab Republic	na *	sildenafil tadalafil	7.08 mg/kg 2.1 mg/kg	0.00708 mg/g 0.0021 mg/g
Herbal honey-based paste from Turkey	na *	sildenafil	290 ± 100 mg/100 g	2.9 mg/g
Herbal honey-based paste from Turkey	na *	sildenafil	3.72 mg/g	3.72 mg/g

* not available.

## Data Availability

The original data presented in the study are openly available in https://webgate.ec.europa.eu/rasff-window/screen/search (accessed on 1 July 2026).

## References

[1] Nemr M.T.M., Abdelaziz M.A., Teleb M., Elmasry A.E., Elshaier Y.A.A.M. (2025). An overview on pharmaceutical applications of phosphodiesterase enzyme 5 (PDE5) inhibitors. Mol. Divers..

[2] Gong B., Ma M., Xie W., Yang X., Huang Y., Sun T., Luo Y., Huang J. (2017). Direct comparison of tadalafil with sildenafil for the treatment of erectile dysfunction: A systematic review and meta-analysis. Int. Urol. Nephrol..

[3] Ala M., Mohammad Jafari R., Dehpour A.R. (2021). Sildenafil beyond erectile dysfunction and pulmonary arterial hypertension: Thinking about new indications. Fundam. Clin. Pharmacol..

[4] Hatzimouratidis K. (2006). Sildenafil in the treatment of erectile dysfunction: An overview of the clinical evidence. Clin. Interv. Aging.

[5] Lan T.-Y., Chiang C.-H., Chen J.-W., Chang T.-T. (2025). Potential beneficial impacts of tadalafil on cardiovascular diseases. J. Chin. Med. Assoc..

[6] Burnett A.L., Nehra A., Breau R.H., Culkin D.J., Faraday M.M., Hakim L.S., Heidelbaugh J., Khera M., McVary K.T., Miner M.M. (2018). Erectile Dysfunction: AUA Guideline. J. Urol..

[7] Arafat S.M.Y., Hossain S., Uddin M.R. (2025). Postorgasmic Side Effects of Tadalafil: A Case Report. Clin. Case Rep..

[8] Kosić-Vukšić J., Krivohlavek A., Žuntar I., Pocrnić M., Galić N. (2024). Undeclared phosphodiesterase type 5 inhibitors (PDE5Is) in food supplements on the Croatian market analyzed by liquid chromatography time-of-flight mass spectrometry (LC-QTOF-MS). Microchem. J..

[9] European Union (2002). Council Directive 2001/110/EC of 20 December 2001 relating to honey. Off. J. Eur. Union.

[10] European Union (2002). Directive 2002/46/EC of the European Parliament and of the Council of 10 June 2002 on the approximation of the laws of the Member States relating to food supplements. Off. J. Eur. Union.

[11] European Union (2002). Regulation (EC) No 178/2002 of the European Parliament and of the Council of 28 January 2002 laying down the general principles and requirements of food law, establishing the European Food Safety Authority and laying down procedures in matters of food safety. Off. J. Eur. Union.

[12] RASFF Window. https://webgate.ec.europa.eu/rasff-window/screen/search.

[13] Vida V., Feketéné Ferenczi A. (2023). Trends in honey consumption and purchasing habits in the European Union. Appl. Stud. Agribus. Commer..

[14] Palma-Morales M., Huertas J.R., Rodríguez-Pérez C. (2023). A Comprehensive Review of the Effect of Honey on Human Health. Nutrients.

[15] Ullah S., Ullah Khan S., Saleh T.A., Fahad S. (2018). Mad honey: Uses, intoxicating/poisoning effects, diagnosis, and treatment. RSC Adv..

[16] Gami R., Dhakal P. (2017). Mad Honey Poisoning: A Review. J. Clin. Toxicol..

[17] Khan I.A., Khan M. (2025). A Fascinating Tale of Mad Honey: No Longer Enigmatic. Proc. Int. Acad. Sci..

[18] European Union (2006). Regulation (EC) No 1925/2006 of the European Parliament and of the Council of 20 December 2006 on the addition of vitamins and minerals and of certain other substances to foods. Off. J. Eur. Union.

[19] European Union (2017). Regulation (EU) 2017/625 of the European Parliament and of the Council of 15 March 2017 on official controls and other official activities performed to ensure the application of food and feed law, rules on animal health and welfare, plant health and plant protection products, amending Regulations (EC) No 999/2001, (EC) No 396/2005, (EC) No 1069/2009, (EC) No 1107/2009, (EU) No 1151/2012, (EU) No 652/2014, (EU) 2016/429 and (EU) 2016/2031 of the European Parliament and of the Council, Council Regulations (EC) No 1/2005 and (EC) No 1099/2009 and Council Directives 98/58/EC, 1999/74/EC, 2007/43/EC, 2008/119/EC and 2008/120/EC, and repealing Regulations (EC) No 854/2004 and (EC) No 882/2004 of the European Parliament and of the Council, Council Directives 89/608/EEC, 89/662/EEC, 90/425/EEC, 91/496/EEC, 96/23/EC, 96/93/EC and 97/78/EC and Council Decision 92/438/EEC (Official Controls Regulation) (Text with EEA relevance). Off. J. Eur. Union.

[20] European Union (2024). Regulation (EC) No 396/2005 of the European Parliament and of the Council of 23 February 2005 on maximum residue levels of pesticides in or on food and feed of plant and animal origin and amending Council Directive 91/414/EEC. Off. J. Eur. Union.

[21] European Union (2009). Regulation (EC) No 470/2009 of the European Parliament and of the Council of 6 May 2009 laying down Community procedures for the establishment of residue limits of pharmacologically active substances in foodstuffs of animal origin, repealing Council Regulation (EEC) No 2377/90 and amending Directive 2001/82/EC of the European Parliament and of the Council and Regulation (EC) No 726/2004 of the European Parliament and of the Council. Off. J. Eur. Union.

[22] Kędzierska-Matysek M., Teter A., Skałecki P., Topyła B., Domaradzki P., Poleszak E., Florek M. (2022). Residues of Pesticides and Heavy Metals in Polish Varietal Honey. Foods.

[23] Morariu I.-D., Avasilcai L., Vieriu M., Lupu V.V., Ioniuc I., Morariu B.-A., Lupu A., Morariu P.-C., Pop O.-L., Burduloi V.M. (2024). A Comprehensive Narrative Review on the Hazards of Bee Honey Adulteration and Contamination. J. Food Qual..

[24] Hudson A., Axon A., Stoneley A., Kane C., French E., Adams L., Smythe L., Iheozor-Ejiofor P. (2024). Honey Risk Profile. FSA Res. Evid..

[25] Asharari K.S., Alali N.M., Magliyah M.S., Al-Dhibi H.A., Almarek F.A., AlBalawi H.B. (2021). Central Serous Chorioretinopathy Following Oral Use of Adulterated Honey Mixed with Tadalafil: A Case Report. Int. Med. Case Rep. J..

[26] Eiden C., Laureau M., Richeval C., Arnal T., Ghomrani H., Peyrière H., Gaulier J.-M., Sebbane M. (2022). Acute cardiovascular disorders related to aphrodisiac honey (“Jaguar power”) consumption: Warning of unintentional exposure to sildenafil. Rev. Med. Interne.

[27] Thiebot P., Magny R., Langrand J., Houzé P., Labat L. (2023). Surdosage en tadalafil par consommation de miel aphrodisiaque vendu sur internet. Toxicol. Anal. Clin..

[28] Schwartz B.G., Kloner R.A. (2010). Drug Interactions With Phosphodiesterase-5 Inhibitors Used for the Treatment of Erectile Dysfunction or Pulmonary Hypertension. Circulation.

[29] Lui J.L., Shaw N.M., Abbasi B., Hakam N., Breyer B.N. (2023). Adverse reactions of PDE5 inhibitors: An analysis of the World Health Organization pharmacovigilance database. Andrology.

[30] Petric Z., Žuntar I., Putnik P., Bursać Kovačević D. (2021). Food–Drug Interactions with Fruit Juices. Foods.

[31] Jairoun A.A., Al-Hemyari S.S., Shahwan M., Zyoud S.H., Ibrahim B., Zyoud S.H. (2022). Screening and Determination of Synthetic PDE-5 Inhibitors in Adulterated Sexual Enhancement Supplements. Molecules.

[32] Sirhan A.Y., AlRashdan Y., Abbasi N.U., Mostafa A., Abudayeh Z., Talhouni A., Al-Ebini Y. (2023). Optimization and Validation of HPLC-UV Method for the Determination of Vardenafil, Sildenafil, and Tadalafil in Honey-Mixed Herbal Sachets Using a Design of Experiment. Jordan J. Pharm. Sci..

[33] Alkhalaf A.M., Alenazi F.K., Abudahash M.M., Alfadhli R., Altoum G.H., Alenzi N.D. (2024). Counterfeiting honey with phosphodiesterase inhibitors for sexual activity enhancement: Detection using high-performance liquid chromatography coupled with a photodiode array detector (HPLC-PAD). J. Men’s Health.

[34] Blokland P., Reniers G. (2020). Safety Science, a Systems Thinking Perspective: From Events to Mental Models and Sustainable Safety. Sustainability.

[35] Pujol C., Danoun S., Biasini G., Retailleau E., Masson J., Balayssac S., Gilard V. (2024). Benchtop NMR Coupled with Chemometrics: A Workflow for Unveiling Hidden Drug Ingredients in Honey-Based Supplements. Molecules.

